# Experience with speech sounds is not necessary for cue trading by budgerigars (*Melopsittacus undulatus*)

**DOI:** 10.1371/journal.pone.0177676

**Published:** 2017-05-31

**Authors:** Mary Flaherty, Micheal L. Dent, James R. Sawusch

**Affiliations:** Department of Psychology, University at Buffalo, The State University of New York, Buffalo, New York, United States of America; Rutgers The State University of New Jersey, UNITED STATES

## Abstract

The influence of experience with human speech sounds on speech perception in budgerigars, vocal mimics whose speech exposure can be tightly controlled in a laboratory setting, was measured. Budgerigars were divided into groups that differed in auditory exposure and then tested on a cue-trading identification paradigm with synthetic speech. Phonetic cue trading is a perceptual phenomenon observed when changes on one cue dimension are offset by changes in another cue dimension while still maintaining the same phonetic percept. The current study examined whether budgerigars would trade the cues of voice onset time (VOT) and the first formant onset frequency when identifying syllable initial stop consonants and if this would be influenced by exposure to speech sounds. There were a total of four different exposure groups: No speech exposure (completely isolated), Passive speech exposure (regular exposure to human speech), and two Speech-trained groups. After the exposure period, all budgerigars were tested for phonetic cue trading using operant conditioning procedures. Birds were trained to peck keys in response to different synthetic speech sounds that began with “d” or “t” and varied in VOT and frequency of the first formant at voicing onset. Once training performance criteria were met, budgerigars were presented with the entire intermediate series, including ambiguous sounds. Responses on these trials were used to determine which speech cues were used, if a trading relation between VOT and the onset frequency of the first formant was present, and whether speech exposure had an influence on perception. Cue trading was found in all birds and these results were largely similar to those of a group of humans. Results indicated that prior speech experience was not a requirement for cue trading by budgerigars. The results are consistent with theories that explain phonetic cue trading in terms of a rich auditory encoding of the speech signal.

## Introduction

Phonetic cue trading experiments on human speech have shown that listeners are able to perceive the same phoneme under a variety of cue manipulations so that, despite changes in the acoustic signal, the same phonetic percept exists. In these experiments, one cue is attenuated and another cue takes over to become the salient cue used to decipher the target phoneme [1]. Relevant cues for a particular phonetic distinction can be defined as any acoustic properties that systematically co-vary with that phonetic distinction. Removing one cue and replacing it with a different cue will not alter the phonetic percept. Cue trading has been cited as being one of the most compelling sources of evidence for an articulation-based speech mode of perception because several early studies observed cue trading between specific spectral and temporal auditory cues occurring only when listeners engaged in phonetic classification of speech signal and not when they were identifying analogous nonspeech stimuli [2]. Based on these findings, phonetic trading relations were believed to be a product of phonetic categorization, specific to speech and reliant on reference to speech production, rather than a result of more general interactions in the auditory system.

An example of trading relations can be observed in the integration of cues for syllable-initial stop consonants. In English, there is a voicing distinction between stop consonants. Voice onset time (VOT) is the primary difference between voiced and voiceless stop consonants (b-p, d-t, and g-k), especially when these consonants are present in the syllable-initial position of the utterance. VOT contains a variety of acoustic information for the listener and plays an important role in the perception of the syllable initial stops [3,4]. Listeners do not attend exclusively to VOT in judging stops in syllable-initial position, however. Other cues have also been found that influence the location of the VOT boundary for speech. One major group of secondary cues to voicing includes the cues that are present in the formants [5–7]. During voicing, there are concentrations of energies that occur in specific frequency ranges, known as formant frequencies, that transition during production, especially at the release of a stop consonant. The duration and extent of formant transitions and their frequency at onset can provide useful cues to the listener. Of interest to the current research are the cues provided by the onset frequency of the first formant (F1).

The amount of time between stop release and the onset of vocal fold vibration results in a variety of consequences in the vocal tract. The delay of voicing onset allows time for the vocal tract to prepare for articulation of the following vowel and therefore causes the first formant frequency to be higher once voicing begins. In voiced stops, voicing begins with little to no delay after release and subsequently leads to a lower first formant frequency at onset, since there has not been sufficient time for the articulators in the vocal tract to transition into the following vowel. This results in a correlated set of acoustic qualities that could cue the voicing percept, where short VOTs and a low first formant onset co-occur, and longer VOTs and a higher first formant onset co-occur.

Due to the correlation between these cues, the onset frequency of F1 has been shown to exhibit a cue trading relationship with VOT [8]. That is, the higher the F1 onset frequency, the shorter the VOT needs to be to perceive a voiceless stop. Since voiceless stops tend to have a higher F1 onset frequency in comparison with voiced stops, both cues can be used interchangeably by listeners to differentiate the voicing contrast [8].

While the existence of cue trading was established decades ago, the mechanism behind cue trading in speech perception is not yet understood. Two general classes of theories have attempted to explain the potential mechanisms involved. One class of theories focuses on a mechanism that makes reference to the articulatory gestures of the vocal tract that are involved in speech production [9–12]. These theories propose that the gestures involved in speech production, such as the coordinated movements of the tongue, jaw and lips, map onto perceived speech. These gestures are the objects of speech perception, which differ from the objects of general audition. Therefore, according to this class of theories, cue trading relies on speech production, from years of articulatory experience or as the result of a perceptual system that is grounded in a reference to speech production. Many of these theories also hold that cue trading is a speech-specific phenomenon processed by a specialized speech mechanism. According to these speech-specific views, listeners are capable of phonetic cue trading by referring to the language-specific properties of the human vocal tract. While trading relations may occur in other modalities, phonetic cue trading in speech is a special type of perception only possible because of the listener’s knowledge of their own production.

The other class of theories states that cue trading occurs via a general auditory mechanism capable of exploiting multiple acoustic cues to identify or categorize all complex sounds, including speech [13,14]. According to this approach, the processes involved in speech perception are the same processes involved in the perception of other auditory events and no specialized speech module or reference to articulatory gestures is necessary. For each piece of evidence in favor of the articulation-based approach [9, 10, 12], there is a corresponding piece of evidence against it [13–16].

One way to uncover whether the mechanism is one that is innate and speech specific (that depends on articulatory gestures) or one that relies on a general auditory process that does not refer to articulation is to consider the role of experience in the discrimination of nonspeech contrasts in humans. A large portion of support for articulation-based theories comes from experiments that highlight the differences between speech and nonspeech perception, which argue that the perceptual system has different modes for perceiving speech versus nonspeech [17–21]. Cue trading has been touted as one example of a “speech-specific” phenomenon. Best and colleagues [17] were among the first to provide evidence for this. First, they demonstrated cue trading between silent gap duration and F1 onset frequency in the perception of “say” versus “stay”. Identification functions were obtained for two synthetic “say-stay” continua, each containing systematic variations in the amount of silence after the /s/ noise. One continuum had a low F1 onset frequency and the other had a high F1 onset frequency. For items with a lower F1 onset frequency, if the silent gap was short (less than 50 ms) listeners reported hearing “say”. If the gap was longer (more than 50 ms), then listeners reported hearing “stay”. For the higher F1 onset frequency items, the silent gap needed to be approximately 24 ms longer for listeners to report hearing “stay”. This exchange between silent gap and F1 frequency is an example of cue trading. To determine if the trading relationship between these two cues would be found with nonspeech, they constructed sinewave versions of the stimuli from the prior experiments. Sinewave speech is a type of synthesized speech that is composed of three to four time-varying sinusoids (sinewaves) in place of the formants. It is constructed in a way that replicates natural speech’s frequency and amplitude patterns, but when heard in isolation by a naïve listener, it tends to be unintelligible and not “speech like”. Instead the listener tends to hear only buzzes and chirps. However, if a listener is aware (or instructed) that the sinewave speech is in fact a speech analogue then they can often hear the speech qualities that exist. In the Best et al. study, listeners’ responses in the sinewave experiment differed dramatically depending on whether or not they heard the sinewave stimuli as speech or nonspeech. Listeners who heard the stimuli as speech exhibited the predicted cue trading effect between the two cues (silent gap duration and F1 frequency). The listeners who heard the sinewave stimuli as nonspeech “whistles” or “computer sounds” showed no evidence of cue trading. These “nonspeech” listeners instead attended to either the spectral or the temporal cue, while the speech listeners integrated these cues into a single percept. This was interpreted as indicating that cue trading is phonetic (language specific) and not psychoacoustic in origin.

If speech perception is based on a general auditory mechanism instead of a speech-specific mechanism, then listeners should not show differences between speech and nonspeech perception, unless that difference is due to extensive experience with speech compared to nonspeech. There are several studies illustrating that many of the phenomena initially thought to be specific to speech can and do occur with nonspeech stimuli. For example, studies have shown that with enough training on nonspeech stimuli, individuals are capable of showing similar “speech specific” behaviors with nonspeech [22–23]. Nonspeech stimuli such as musical chords, noise bursts, and slamming doors have been found to elicit labeling behavior similar to what has been shown with speech [13].

Human speech sound perception by nonhuman animals is, in a sense, equivalent to nonspeech sound perception by humans [24]. Research with Japanese quail (*Coturnix japonica*) and other nonhuman animals shows that even with limited or no experience with speech, the perceptual phenomena thought to be specific to speech are still observed [25–29]. Kluender [30] controlled the amount of exposure quail had with VOT stimuli by never presenting intermediate or co-varying stimuli to the quail during training. Limiting the training stimuli ensured that any observed cue trading effects during testing were not due to prior training experience with the co-occurrence of these cues. Despite their lack of direct exposure to these intermediate stimuli, the quail still exhibited cue-trading abilities similar to what has been found in humans. It is important to point out that the quail in this study were exposed to conversational speech within the laboratory setting, so the influence of passive exposure to the co-occurrence of speech cues could not be ruled out. However, nonhuman animals in general do show sensitivity to speech contrasts, often performing better than humans do with nonspeech contrasts [12,31–34]. This could be due to the constraints placed on humans by their native language experience [24]. Just as native language experience influences second language learning in humans, it may also influence human nonspeech perception in a similar way [35–36].

Overall, the cross-language, comparative, and developmental data do not provide conclusive evidence for mechanisms underlying speech perception, or trading relations in particular. Prior comparative data on cue trading ability has been inconclusive due to conflicting results. While Kluender and Lotto [37] showed a psychoacoustic basis for trading relations using a discrimination task with Japanese quail, Sinnott and Saporita [38] were unable to demonstrate cue trading in monkeys (*Macaca fuscata*). There has also been a lack of strict control over exposure to co-varying cues [34,37–39]. In addition, developmental studies with humans have been unable to differentiate between maturation and learning in children and adults [40–41].

The debate regarding the mechanisms of speech perception continues today and has become more popular in light of recent neurophysiological data that suggest recruitment of the motor system during perception of speech [42–44]. The main findings of these studies have focused on mirror neurons in monkeys, which are a class of neurons that respond when a monkey performs or observes an action [45–46]. These mirror neurons appear to provide a one-to-one mapping between perception and action and have therefore gained popularity as evidence for a neural mechanism for linking speech perception to gestures, as proposed by articulation-based theories. These claims are based primarily on data from monkey studies, which did not directly test speech perception. Corresponding studies of mirror neurons in humans contradict many of the claims of articulation-based theories [47–48]. In addition, these studies have not proposed the processes by which the listener would map context-dependent acoustics to motor representations. Despite the contradictory evidence of mirror neurons’ involvement in speech processing, what evidence exists is often cited as support for a new form of articulation-based theories that point to sensorimotor integration as the mechanism behind the perception of speech [9,49–50]. Studies using transcranial magnetic stimulation (TMS) and functional imaging have shown the motor speech system becomes activated during speech perception, even when there is no explicit motor task involved [51–52]. These findings suggest that the motor system may modulate speech perception, but there is no direct evidence that these activations are necessary for speech perception as the articulation-based theories would claim [47].

The purpose of this research was to investigate the potential mechanisms underlying speech perception using a population that allows for greater control of vocal and perceptual learning. By using budgerigars (*Melopsittacus undulatus*) as subjects, we are able to control speech experience and exposure from birth and test the effects of these differences. A large amount of research exists showing that these animals are ideal subjects for psychoacoustic studies, due to their highly sophisticated and flexible perceptual learning system [53]. While there has been no investigation of how specific speech training or experience may influence their perceptual abilities, budgerigars have been used in a variety of studies investigating their hearing and their perceptual abilities. As a vocal mimic, these birds are an interesting subject to investigate. Additionally, they are capable of perceiving both consonant and vowel tokens in a manner very similar to humans [53–56].

To date, no studies have been conducted that investigate cue trading by budgerigars or the influence that human speech experience may have on the phenomenon. As vocal mimics, budgerigars were an ideal candidate for this study and permitted investigation of the role articulatory training plays in speech perception and whether or not any prior exposure to speech can influence cue-trading behaviors with our current phonetic contrast. By tightly controlling speech experience from birth, the current study compared perception of speech sounds and the presence of cue trading across four groups of budgerigars. There was also a group of human participants tested for comparison. The budgerigars were divided into four groups prior to hatching. One group was completely isolated from human speech sounds, one group was passively exposed to conversational speech sounds, and two groups were extensively trained to mimic human speech sounds. One of these speech-trained groups was trained to mimic syllables containing the target phonemes (/d/ and /t/) that they would later be tested on, while the other speech-trained group was trained to mimic non-target phonemes that would not be included in later testing. The goal was to determine whether these differences in experience would influence cue trading by the birds in synthetic /da/-/ta/ continua.

If experience with human speech alters cue trading behavior in birds, then the birds with human speech experience should show different results than those without speech experience. If production is a prerequisite for cue trading, then only the birds capable of mimicking the human speech should show the effect. Alternatively, if extensive exposure to a particular speech contrast is necessary, then the birds trained on the target phonemes should show different results than the birds trained on the non-target phonemes. If cue trading relies on a general auditory mechanism that does not depend on experience, then there should be no differences between any of the groups. Comparisons with humans will reveal the extent of the similarities and differences between the species.

## Materials and methods

### Subjects

This study included 25 human participants as subjects, ranging in age from 18 to 21 years, with a mean of 18.8 years. All 25 participants were undergraduate students from an introductory psychology course at the State University of New York at Buffalo who participated in the experiment for course credit. All were required to be native speakers of English with no reported history of a speech or hearing disorder. During debriefing we discovered two of our listeners were not native speakers of English. Their data were not included in the analysis.

This study also included 25 budgerigars (13 males, 12 females). Eleven of the birds were housed in a vivarium with a normal bird population that were not participating in the present study, and fourteen of the birds were individually housed in sound-attenuated isolation chambers at the University at Buffalo. Both rooms were kept on a day/night cycle corresponding to the season. All birds were kept at approximately 90% of their free-feeding weight during the testing phase of the experiment. Controlled exposure to speech sounds was initiated during the breeding process and continued for the duration of the experiment. Regardless of rearing condition, all birds had regular contact with humans each day. Speech training for birds in the two speech training groups began after fledgling and took place 1–2 hours per day, 5–6 days per week. After approximately 6 months, perceptual testing began for all groups. Testing sessions took place twice a day, 5–7 days a week and lasted 30–40 minutes per session. All procedures were approved by the University at Buffalo, SUNY’s Institutional Animal Care and Use Committee and complied with NIH guidelines for animal use.

### Testing apparatus for bird subjects

The experiments took place in one of four identical psychoacoustic testing setups. The setups consisted of a wire test cage (61 x 33 x 36 cm) mounted in a sound-attenuated chamber (Industrial Acoustics Company, Small Animal Chamber) lined with sound-absorbent foam (10.2 Sonex, Ilbruck Co.). The test cage contained a perch, an automatic food hopper (Med Associates Standard Pigeon Grain Hopper), and two vertical response keys extending downwards from the inside of the hopper in front of the perch. The response keys were two sensitive microswitches with 1 cm^2 green (left key) or red (right key) buttons glued to the ends. The microswitches were tripped when the birds pecked the colored keys. A small 7-W light at the top of the test cage illuminated the chamber and served as the experimental house light. An additional 30-W bulb remained on in the chamber for the entire session. The behavior of the animals was monitored at all times during testing by an overhead web-camera (Logitech QuickCam Pro, Model 4000). One speaker (Morel Acoustics, Model MDT-29) hung directly above the bird’s head, 18 cm away from the bird during testing. The experiments were controlled by a Dell microcomputer operating Tucker-Davis Technologies (TDT, Gainesville, FL) modules and SykofizX software.

### Testing apparatus for human subjects

Human participants were tested individually in a room within a human speech research laboratory. An Apple computer controlled stimulus presentation and response collection. The stimuli were presented binaurally through headphones (TDH-39, Telephonics). Listeners were asked to identify the initial phoneme as /d/ or /t/ by pressing one of two buttons on a computer-controlled response box. The speed of presentations was dependent upon the listener’s response speed. The next stimulus was presented 1 s after the listener responded or after 4 s from syllable onset, whichever came first. The listeners’ response and reaction time (RT) were recorded for each stimulus.

### Stimuli

The stimuli were two series of five-formant /da/-/ta/ syllables generated using the parallel branch of the Klatt [57] software synthesizer implemented on an Apple computer. All stimuli were matched for peak amplitude and total duration (250 ms). The two stimulus series differed only in the frequency of F1. The two F1 frequency values were 375 and 750 Hz, modeled after the stimuli used by Kluender and Lotto [37]. The F1 for all stimuli contained no formant transitions, but instead onset at the value described and remained at that steady state value (low or high) throughout each utterance. This manipulation avoids introducing transition durations and transition extent to the stimuli as potential cues to this voicing contrast [7]. Within each series there were eight stimuli differing only in voice onset time (VOT). Change from initial voiced to initial voiceless stop was accomplished by changing the VOT from 5 to 75 ms in 10 ms steps. Variation of the VOT was achieved by delaying the onset of energy at F1 relative to the onset of the higher formants and by exciting F2 and F3 with a noise source instead of the periodic glottal source during the period of delay between release and voicing.

**Fig 1:**
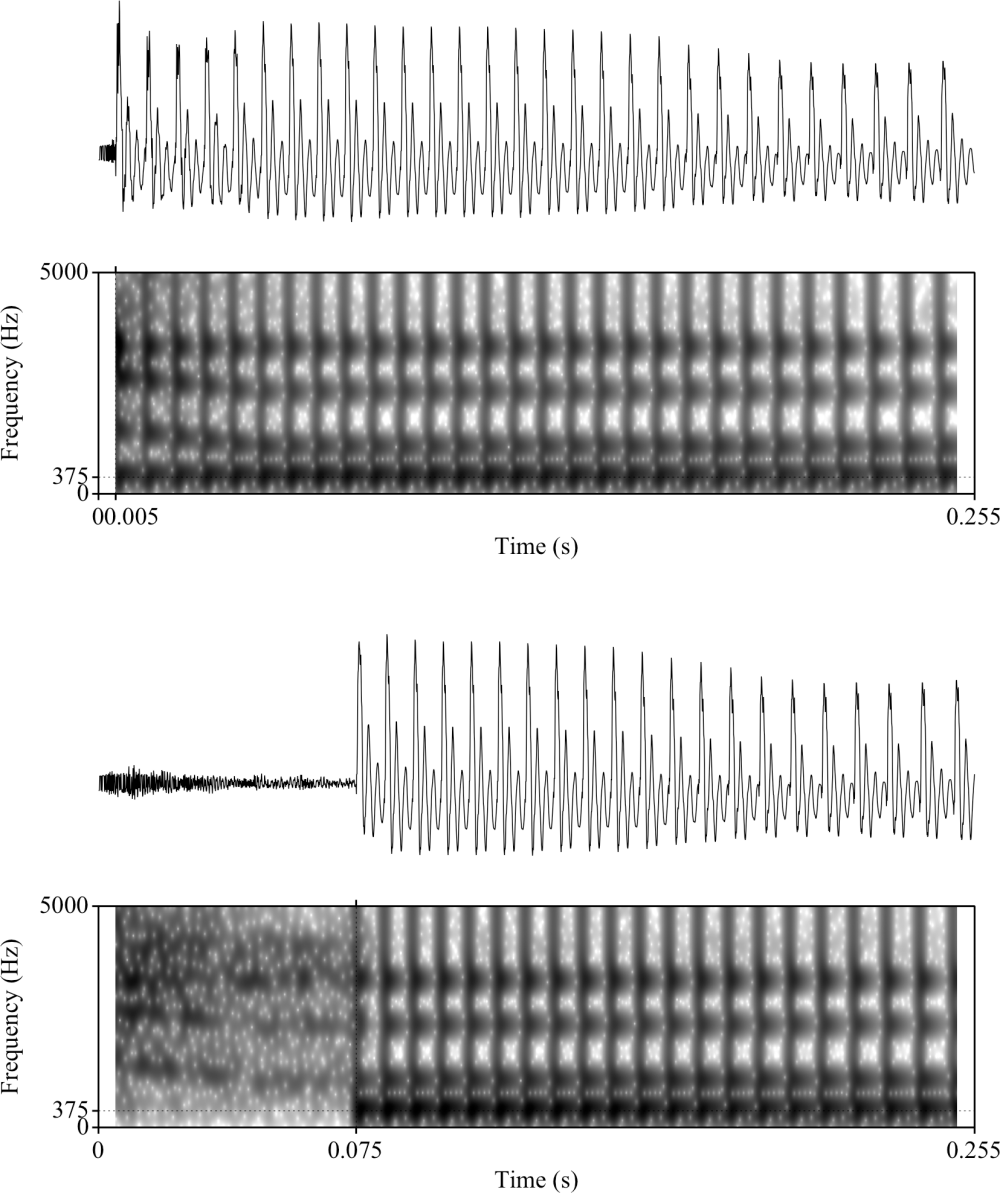
Spectrograms of synthetic speech syllables with low F1 frequency, "da" (upper box) and "ta" (lower box). The F1 frequency for both syllables at the onset of voicing is 375 Hz. Both stimuli are 255 ms in duration.

**Fig 2:**
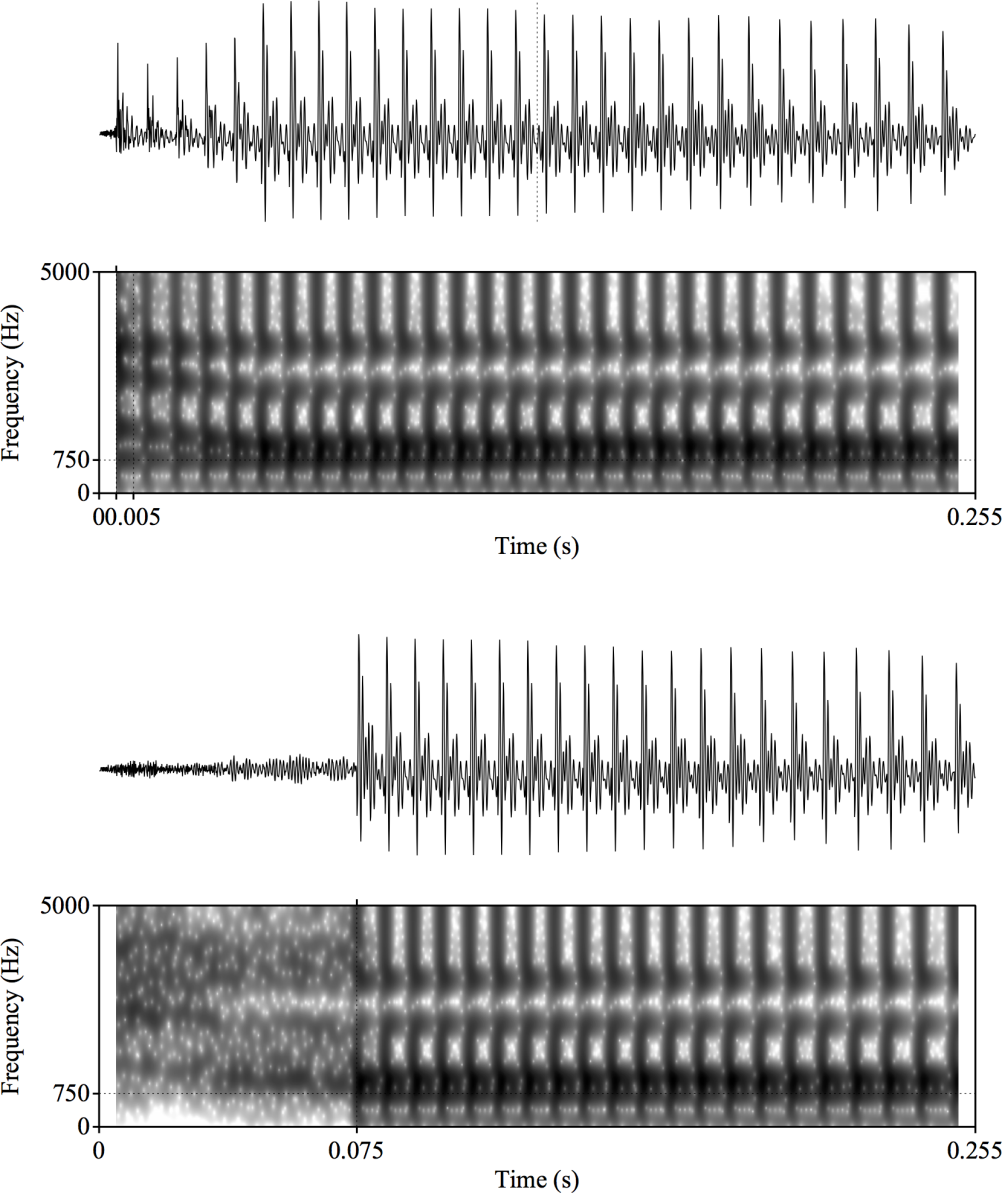
Spectrograms of synthetic speech syllables with high F1 frequency, "da" (upper box) and "ta" (lower box). The F1 frequency for both syllables at the onset of voicing is 750 Hz. Both stimuli are 255 ms in duration.

For this alveolar series, F2 began at 1950 Hz and fell linearly to 1220 Hz over 40 ms while F3 fell from 3000 to 2600 Hz. The fourth and fifth formants were flat at values of 3300 and 3850 Hz. The series was based on analysis of natural /da/ and /ta/ utterances as spoken by an adult male, author JRS. Spectrogram examples of the synthetic stimuli endpoints for both the high and low F1 frequency are shown in Figs 1 and 2. Fig 1 depicts two of the endpoints for the low F1 frequency (375 Hz) items, with a 5 ms VOT (“da”) in the top spectrogram and a 75 ms VOT (“ta”) in the bottom spectrogram. Fig 2 depicts two of the endpoints for the high F1 frequency (750 Hz) items, with a 5 ms VOT (“da”) in the top spectrogram and a 75 ms VOT (“ta”) in the bottom spectrogram. Stimuli were synthesized with 16-bit resolution at a 10-kHz sampling rate. The same stimuli were presented to both human and non-human subjects.

### Training and testing procedures for budgerigars

Birds were trained using operant conditioning procedures to peck left and right spatially separated microswitches for food reinforcement. The birds were first trained to distinguish between two pure tones (2 kHz vs. 3 kHz). Training birds to categorize the two tones reduces their exposure to speech stimuli prior to testing. To start a trial, the birds were trained to peck the left key, activating a variable waiting interval of 2–7 s. After this variable interval, one of the two tone stimuli was presented from the loudspeaker. If they correctly classify the tone by pecking the appropriate response key (for some birds, left for “2 kHz” and right for “3 kHz”, for other birds, left for “3 kHz” and right for “2 kHz”), they were reinforced with 1.5 s access to hulled millet from the illuminated food hopper for 70% of the correct trials. They were reinforced with only the hopper light for 1.5 s in the other 30% of the correct trials. If they responded incorrectly, the hopper light was extinguished for 5 s. A training criterion performance of at least 80% correct for 300 consecutive trials was required before moving to the next phase of training. Once criterion was reached, the birds were trained to distinguish between synthetic speech stimuli of two series with VOTs of 5 ms and 15 ms (for the voiced end of the continuum) and 65 and 75 ms (for the voiceless end of the continuum). The two series differed from one another only in their F1 onset frequency values (375 and 750 Hz). The training procedure was identical to the tone training except that the F1 onset frequency varied on alternate days. Once training criterion performance of at least 80% correct for 300 consecutive trials was reached, experimental testing was started. During experimental testing, the procedure was identical except intermediate VOT stimuli were presented to the birds in 20% of the trials and food reinforcement was provided regardless of how they categorized the intermediate stimuli. All birds completed a minimum of 200 trials per condition. During all training and testing, continua were alternated daily between the low F1 onset and the high F1 onset continua.

There were four “rearing” conditions, with a minimum of five birds in each condition: (1) auditory isolation condition, (2) passive auditory condition, (3) target-phoneme speech trained condition, and (4) non-target phoneme speech trained condition. Housing conditions for each bird are presented in Table 1. Ten of the birds were individually housed in wire cages in the same room where birds are usually housed (passive auditory condition and some speech trained condition birds). Fourteen birds were individually housed in specially constructed Lucite cast acrylic cages, similar in size to the wire cages housing the other birds. The acrylic cages were equipped with ventilation fans to ensure proper ventilation but otherwise were identical in set up to the wire cages (containing perches, toys, cuttlebone, and food and water dishes). The purpose of these cages was to attenuate any outside human speech sounds. The fourteen acrylic cages were placed in a different room from the rest of the bird population. This room contained white noise machines designed to mask any outside human speech sounds, which continuously played at a level of approximately 70 dB SPL at all times. 70 dB is about 5 dB above “conversational speaking level” of humans. The white noise was required for any human speech sounds in the hallway to not reach the birds. The walls of this room were lined with sound-attenuating foam attached to large wooden boards for easy cleaning and replacing. This procedure using acrylic cages and speech isolation continued for the duration of the experiment.

**Table 1:** Housing type (sound attenuated vs normal vivarium) for individual birds within each exposure group.

	Number of Birds	Number in Sound Attenuated Room (acrylic isolate cage)	Number in Normal Vivarium (wire cage)
Speech Isolated	6	6	0
Passive Listening	7	0	7
Non-Target Speech Trained	5	4	1
Target Speech Trained	7	4	3
Total	25	14	11

The majority of the budgerigars used in these experiments were bred in the laboratory for the purposes of the experiment. Three of the birds were acquired from a local pet store. These birds were placed in the passive auditory condition. All of the birds in the auditory isolation condition and 8 birds from the speech-trained conditions were bred and raised in the sound-attenuated room. The remaining 4 birds from the speech-trained conditions and all of the birds in the passive auditory condition were raised in the regular vivarium. Once the birds were fledged and eating regularly (as noted by their weights), they were individually housed in wire or acrylic cages.

Of the fourteen birds housed in the acrylic cages, six were completely isolated from all human speech sounds (auditory isolation condition). The other eight birds were taken outside of the room daily to be individually trained to produce human speech sounds. To do this training, we used the model-rival procedure described by Banta and Pepperberg [58]. Between 5–7 days a week from when the birds were 2 months old until approximately 7 months old, the birds were placed in a carrying cage and transported to a training room once or twice a day for approximately 60 minutes per session. During training sessions, the birds were placed on a perch atop a desk between two human experimenters. The experimenters repeated human words as in the model-rival paradigm, attempting to teach the budgerigars human speech sounds. The birds were returned to their home cages for the non-training periods via the carrying cage. Audio and video recordings were taken of the sessions for later analysis.

There were two speech-trained groups. One group (Target Speech Trained) was trained to produce utterances that contained the stimuli used in the behavioral testing. These words and nonwords were: “dash”, “tash”, “dish”, and “tish”. The other group (Non-target Speech Trained) was trained to produce utterances that contained stimuli that would not be used in the behavioral testing. These nonwords were: “oose”, “eese”, “oosh”, and “eesh”. Once the training, exposure, or isolation periods were complete (after approximately 6 months), all birds were tested on their ability to use phonetic speech cues in the cue trading operant experiments as described above.

### Training and testing procedures for human participants

All stimuli were pilot tested using human participants in order to verify intelligibility and to confirm that the stimuli would elicit the relevant cue-trading effect. Stimuli were presented in block randomized order for both practice and testing. The session began with one practice block and was followed by 4 data blocks. Within each block of trials, all 16 stimuli were presented in random order before any stimulus was repeated. During the data blocks, stimuli were presented in blocks of 80 trials (5 repetitions of each of the 16 items). All listeners participated in 4 blocks of experimental trials, which resulted in a total of 20 responses to each stimulus. During analyses it was discovered that 4 participants failed to consistently label the ends of the series as /d/ and /t/, so their data were not included in the analysis. This resulted in 19 human listeners’ data being used for comparison to the birds.

### Analysis

This study was designed to evaluate the ability of budgerigars to show cue trading behaviors using an alveolar voicing contrast and determine the influence of experience and training on this cue trading behavior. The primary measure of cue trading derived for each participant was the difference in the locations of the /d/-/t/ category boundary (in ms VOT) and the slope of the identification function for each series. The series with the low F1 frequency should have an earlier category boundary (shorter VOTs) than the series with the high F1 frequency, and the slopes should be equivalent. In order to determine the category boundary for each series, the proportion of /d/ response given at each VOT value was tabulated for each listener for each series. These proportions were transformed to probit functions, and from these functions category boundaries and slopes were computed, using the technique proposed by Engen [59] for fitting a psychometric function to a set of data. The fitted function represents a cumulative normal curve. It rests on the assumption that the underlying probability of a particular response is normally distributed. The probability of a /d/ response is converted to a z-score. This transforms the data so that the classification function should be linear with respect to the stimulus dimension (VOT). A regression line can then be fit to the transformed data and the slope of the best fitting line is an estimate of the steepness of the classification (labeling) function. The method is similar to the use of probits described by Finney [60].

In order to measure if there were any differences between the groups in labeling stimuli across the entire series, rather than just at the category boundary, the obtained overall percentage of /d/ responses to all eight stimuli in each series was also analyzed. If listeners exhibit cue trading for VOT and F1 frequency, then the series with a low F1 frequency should yield more overall /d/ responses compared to the series with the high F1 frequency. This analysis includes comparison of differences in labeling responses at the endpoints. This secondary measure allows us to more closely investigate whether exposure group influenced responses for the two series.

## Results

A key element in this study was the attempt to train budgerigars, known vocal mimics, to produce human speech syllables. One group was trained to mimic 4 syllables beginning with the target phonemes and another group was trained to mimic syllables beginning with vowels. The birds received training at least one hour per day, 5–6 days per week for 6 months, and thus were extensively and repeatedly exposed to the syllables that they were trained to mimic. Despite our efforts, there was no evidence that any of the birds were successful mimics of the target or non-target syllables, for either group. Therefore, we do not know to what extent being a vocal mimic may influence performance. Regardless, target contrast trained group *heard* the target phonemes many more times than the other groups (approximately 200 times per day, 5–7 days per week, for 6 months = ~35,000 times), and thus *exposure* to the phonemes differed widely between the groups and could have affected phoneme boundaries and other general abilities to perceive the stimuli.

### Bird group comparison

**Fig 3:**
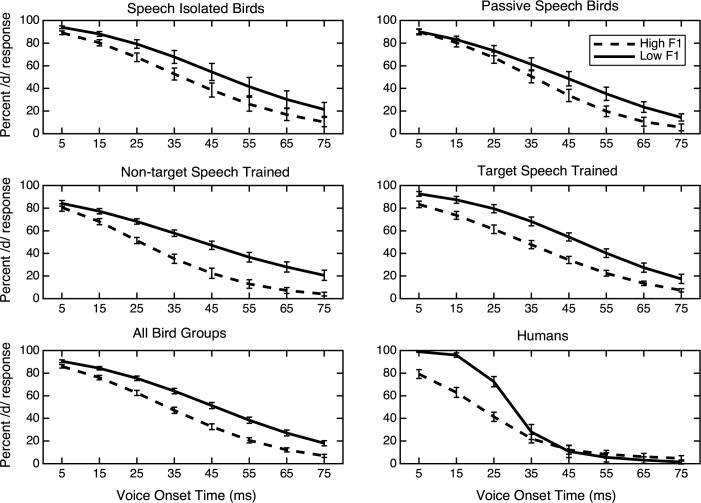
Fitted identification functions showing percentage of /d/ responses for birds and humans across various VOTs. The solid lines represent the responses to the low F1 stimuli, the dashed lines represent responses to the high F1 stimuli. Error bars represent ±1 standard error of the mean.

The fitted identification functions for each group are shown in Fig 3. The graphs show percent identification of the stimuli in each series as /d/, as a function of VOT. The two curves correspond to the two series with the different F1 frequencies. Each graph represents the pooled results for one group of participants. Evidence of cue trading can be seen if the category boundary for VOT is at a higher value (more voiced responses) for the low F1 onset frequency series (solid line) relative to the high F1 onset series (dashed line). This expected cue trading effect was found for all four groups of birds and for the human listeners (see Fig 3A–3D and 3F). There were also no differences in the magnitude of the cue trading effect between any of the exposure groups.

The two-way ANOVA used to assess differences in the location of the category boundaries for the two stimulus series in the four groups of birds with repeated measures on the F1 frequency variable indicated a significant main effect of F1 frequency on the boundaries [*F*(1, 21) = 107.62, *p* < 0.001, $\eta_{p}^{2}$ = 0.837]. This was result of a significant difference in category boundaries between the two series (see Table 2). As a group, the birds had a category boundary at 46.8 ms VOT for the low F1 frequency series compared to a category boundary of 33.3 ms VOT for the high F1 frequency series. This was a trading relation of 13.5 ms VOT. There was no main effect of exposure group [*F*(1, 21) = 1.60, *p* = 0.217], and the interaction between group and series was not significant [*F*(1, 21) = 2.37, *p* = 0.099], indicating the birds’ responses were substantially similar across groups. There were also no significant differences in slopes of the identification functions for either F1 onset frequency, [*F*(1, 21) = 2.65, *p* = 0.118] or exposure group [*F*(3, 21) = 0.92, *p* = 0.450]. Thus, the steepness of the identification functions was statistically equivalent for both series for all groups of birds, regardless of exposure condition.

**Table 2:** Phoneme boundaries (in ms) for the low and high F1 onset *da-ta* continua and the amount of shift in the phoneme boundary (in ms).

	High F1	Low F1	Shift
Speech Isolated	38.4	52.1	13.7
Passive Listening	35.5	43.5	8.0
Non-Target Speech Trained	26.3	43.1	16.8
Target Speech Trained	33.0	48.5	15.5
Humans	21.3	31.8	10.5

The two-way ANOVA used to assess differences in the percentage of /d/ responses for the two stimulus series mirrored that of the category boundary data. There was a main effect of stimulus series, *F*(1,21) = 79.90, *p* = 0.000, $\eta_{p}^{2}$ = 0.792, but no main effect of group [*F*(3,21) = 1.03, *p* = 0.398], and there was no interaction between stimulus series and group [*F*(3,21) = 1.01, *p* = 0.409]. The main effect of stimulus series was a result of a higher proportion of /d/ responses, across the series, when the F1 frequency was low, while fewer /d/ responses were observed when the F1 frequency was high. The birds categorized the high F1 series as /d/ 43.8% of the time and categorized the low F1 series as /d/ 56.2% of the time. That is, the trading relation traditionally observed between VOT and F1 frequency in humans was observed in all of the budgerigars, regardless of exposure to human speech sounds.

### Human data

The humans participants had a trading relation between VOT and F1 frequency of 10.5 ms, which was a significant category boundary difference by one-way ANOVA [*F* (1,18) = 7.09, *p* = 0.016, $\eta_{p}^{2}$ = 0.282]. The human participants had significant difference in percentage of /d/ responses (9.3%) by a one-way ANOVA [*F* (1,18) = 5.03, *p* = 0.038, $\eta_{p}^{2}$ = 0.219]. They categorized the high F1 series as /d/ 31.9% of the time and categorized the low F1 series as /d/ 41.2% of the time. These human data (shown in Fig 3F) are similar to previously reported trading relations between F1 onset frequency and VOT [61–63], indicating a change in category boundary and percent /d/ response as a function of F1 onset frequency. There was also a significant difference in the steepness of the slopes between the two series [*F*(1, 18) = 15.64, *p* = 0.001, $\eta_{p}^{2}$ = 0.465], which is discussed below.

### Human/Budgerigar comparison

The human and bird data were compared using two-way ANOVAs with F1 frequency as a repeated measures factor and participant species (bird or human) as the between groups factor. Since the ANOVA for the bird data showed no effect of training group, all four bird groups were collapsed into a single group for these analyses.

The ANOVA on the category boundary data showed a main effect of F1 onset frequency, *F*(1,42) = 38.35, *p* = 0.000, $\eta_{p}^{2}$ = 0.477. The human participant category boundary data showed a 10.5 ms trading between VOT and F1 frequency and the birds showed a 13.5 ms trading relation. There was also a main effect of species for the category boundary data [*F*(1,42) = 33.68, p = 0.000, $\eta_{p}^{2}$ = 0.445]. Overall, the category boundaries were at longer VOTs for the birds. Finally, no significant interaction between species and F1 onset frequency was found [*F*(1,42) = 0.37, *p* = 0.545)]. This indicates that the magnitude of the trading relation was similar for the birds and the humans.

The ANOVA comparing the percentage of /d/ responses showed a main effect of F1 onset frequency, *F*(1,42) = 30.22, *p* = 0.000, $\eta_{p}^{2}$ = 0.418. There was also a main effect of species for the percentage of /d/ responses [*F*(1,42) = 35.07, *p* = 0.000, $\eta_{p}^{2}$ = 0.455]. There was a higher percentage of /d/ responses overall for the birds compared to the humans. There was no significant interaction between F1 onset frequency and species [*F*(1,42) = 0.61, *p* = 0.440].

In summary, all of the groups showed a significant effect of F1 onset frequency. All birds made more “d” judgments when the F1 onset frequency was low and fewer “d” judgments when the F1 onset frequency was high, consistent with the predicted cue trading pattern. Differences in identification performance and category boundaries for the speech continua did not differ significantly in relation to the speech-exposure group. Human participant data showed a comparable overall pattern. As with the birds, the humans made more “d” judgments when the F1 onset frequency was low and fewer “d” judgments when the F1 onset frequency was high. Overall, category boundaries for humans were at shorter VOTs (fewer “d” responses) when compared to the birds.

### Slope analysis

Visual inspection of the group identification functions for humans (Fig 3F) shows that they appear to be steeper with a more abrupt change in labeling from /d/ to /t/ than those for the birds (Fig 3E). In comparison, the group labeling data for the four groups of birds all appear quite similar to one another. Mean slopes for each group are shown in Table 3. The two-way ANOVA comparing the slope of the labeling function between birds and humans showed a main effect of F1 onset frequency, *F*(1,42) = 17.17, *p* = 0.000, $\eta_{p}^{2}$ = 0.290, indicating a significant difference in slope for the two series. The slope was steeper for the low F1 series. There was also a main effect of participant species, *F*(1,42) = 71.66, *p* = 0.000, $\eta_{p}^{2}$ = 0.630, and a significant interaction between F1 onset frequency and species, *F*(1,42) = 22.25, *p* = 0.000, $\eta_{p}^{2}$ = 0.346. The slope of the identification function was steeper for the humans in the low F1 series, and the function was shallower for the humans in the high F1 series. For the birds, the slope was uniformly shallow for both series.

**Table 3:** Mean slopes in probit units per ms of VOT. (Numbers in parentheses are standard deviations).

	High F1	Low F1
Speech Isolated	-0.40(0.10)	-0.42(0.14)
Passive Listening	-0.47(0.25)	-0.37(0.08)
Non-Target Speech Trained	-0.43(0.16)	-0.27(0.09)
Target Speech Trained	-0.36(0.06)	-0.27(0.09)
Humans	-0.71(0.37)	-1.45(0.72)

## Discussion

This study is the first to provide evidence of cue trading behavior in budgerigars and the first to find cue trading using an identification task in a non-primate species. After learning the categories based on the ends of the two series, all of the birds exhibited cue trading when exposed to the novel, intermediate stimuli of the two series that differed in F1 onset frequency. That is, each budgerigar showed a trading relation between the two acoustic consequences of the articulatory distinction between /d/ and /t/ used in these stimuli. The birds responded /d/ when the VOT was short and /t/ when the VOT was long. There was a shift in the category boundary as a result of the F1 onset frequency, indicating the budgerigars relied on evidence for the distinction from this other acoustic cue. These two cues, VOT and F1 onset frequency, although from different acoustic dimensions, were both exploited by the budgerigars in responding to the two continua and showed a classic trading relation. Similar to Kluender’s work with quail [16], finding this effect in a non-human animal supports the idea that the cue trading phenomenon is not a speech-specific effect as once proposed [18–19,63]. Furthermore, unlike the studies by Kluender, the current study’s strict control of experience ensured there was no opportunity for the birds in the speech isolated exposure group to use experienced covariation of F1 onset frequency and VOT, yet all showed the trading relation, suggesting a robust auditory interaction between these two cues. Prior studies have not definitively ruled out the potential role of passive exposure to laboratory conversational speech.

This is also the first test of the effects of speech exposure on the phenomenon of cue trading. The present research demonstrates that experience with natural speech is not a prerequisite for cue trading behavior by budgerigars. The cue trading behavior was found in all four groups of birds and there were no differences between any of the bird groups. That is, there were no differences between the birds that had experience with human speech sounds and the birds that were completely isolated from all human speech sounds. There were no differences found between birds with extensive speech training and those without any training. Additionally, the content of the speech training sessions did not have any impact on cue-trading behavior. Birds that were trained to mimic syllables beginning with the target phonemes did not differ from the birds that were trained to mimic vowel-initial syllables. While none of the birds ever produced the intended syllables (at least during our training sessions), those in the speech training groups received individualized training sessions that exposed them extensively to the target or non-target phonemes. This substantial amount of experience with these syllables did not lead to a difference in responses compared to the groups without this experience. This indicates that exposure to natural speech is not required to exploit the cues present in the speech signal and there is no advantage of being repeatedly exposed to the target phonemes for these budgerigars. While negative results such as these need to be interpreted with caution, they indicate that the basic phenomena of cue trading for F1 onset and VOT does not require prior exposure. The isolated birds, having had no exposure to prior speech, could not use a specialized speech mechanism to distinguish between these sounds. The birds, having never successfully produced the speech sounds themselves, could not be using an articulatory-based mechanism. Instead, these results provide evidence consistent with the presence of a general auditory mechanism in budgerigar speech perception that is not reliant on speech experience or articulatory knowledge. Thus, at least for the budgerigars, speech categories are either innate or learned auditory categories, processed by the same auditory mechanism that processes other sounds in the environment.

If these results are interpreted as excluding a phonetic, articulatory-based mechanism, two other possible mechanisms remain. One is an innate mechanism that operates purely on psychoacoustic principles that do not need to be learned. The other is a learning mechanism based on general association principles that are not reliant on specific exposure to language. The current results support a psychoacoustic mechanism over a learning mechanism since the birds in the isolated condition of this experiment showed the predicted cue trading behavior between F1 onset and VOT even though they never heard human speech or the covariation of the relevant speech cues until they were exposed in the operant testing booths during the course of the experiments. The birds exploited perceptual patterns and acoustic variations in the stimuli without making any reference to speech knowledge. That the birds were able to use the stimulus characteristics without prior experience suggests that the auditory system may be hard wired to exploit available cues in the physical properties of the stimuli. Following Diehl and Kluender’s [64] auditory enhancement theory, perception of voiced stops may be enhanced by lower formant frequencies through perceptual interactions that result from the operating characteristics of the auditory system. The covariation of these cues may occur because they provide a perceptual advantage to the listener. This could suggest that speech evolved in response to these prewired abilities of the auditory system [65].

These findings are in opposition to articulation-based perception theories that would suggest the perceptual integration of these two cues is based on properties of vocal tract functioning or specialized neural mechanisms that make reference to articulation during perception. The perceptual integration of VOT and F1 onset frequency indicate that the birds treated these cues as containing comparable information based on psychoacoustic factors. The auditory system provides a rich encoding of the information that can be readily used. Articulatory consequences are not a prerequisite for trading relations, at least in birds.

The results from the human participants in this study replicate previous research showing a cue trading relationship between F1 onset frequency and VOT values [61–62]. Their responses had a pattern similar to the budgerigars’ responses. Low F1 onset frequency resulted in more /d/ responses, while high F1 onset frequency resulted in more /t/ responses, especially for shorter VOT values. Unlike the birds, the human participants did not receive any training on the synthetic speech stimuli prior to testing. Some of the listeners had difficulty identifying the endpoints of the series, as is often seen for synthetic speech stimuli [17]. In particular, humans had difficulty classifying the high F1 onset frequency stimuli that had shorter VOT values. Four human participants’ data had to be excluded from the study because they failed to meet the two-category criterion. For these participants, the high F1 onset frequency series stimuli were all classified as voiceless (there was no /d/ category, only /t/ responses to all stimuli in that series). This is likely because naturally produced utterances with short VOT values do not normally have high F1 frequency at onset and therefore the co-occurrence of those cues was unfamiliar to the listeners. Despite these difficulties, the human participants showed the trading relation between F1 onset frequency and VOT values.

The pattern of responses was similar for budgerigars and humans in that both groups had more /d/ responses in the high F1 onset frequency compared to the low F1 onset frequency and both showed the expected shift in category boundary. The cue trading effect in humans was similar to the effect in budgerigars. In humans, the category boundaries between the two series differed by 10.5 ms VOT compared to 13.5 ms VOT for the budgerigars. There was a difference in the slope of the identification functions between the species, with humans showing a steeper slope than the birds for both series (see Table 3). The steepness of the slope, which shows the change in probit units per milliseconds of VOT, generally provides a measure of the degree of category belongingness of the individual response curves, or the extent to which listeners responses are based on VOT. The steeper slopes could, in part, be due to the extensive experience humans have with natural speech. Unlike the budgerigars, the human participants in this study have extensive experience with the correlated nature of VOT and F1 onset frequency cues in natural speech. This extensive experience with speech could lead to them having a greater reliance on VOT compared to the budgerigars. The birds having no experience with either cue would be more likely to use both cues equally, so the shallower functions of the birds likely indicate that the VOT had less weight for the birds than the humans.

The current research, in addition to other studies with nonspeech and other nonhuman animals, supports the hypothesis that perceptual equivalence observed in cue trading is not a characteristic of speech perception but is a general quality in the perception of complex acoustic information for naturally occurring auditory contrasts [16,38]. The auditory system is sensitive to both spectral and temporal contrasts. The current findings provide strong evidence in favor of a general auditory mechanism behind the integration and use of speech cues in distinguishing between alveolar stop consonants, and speech perception in general.

To assess whether speech experience or articulation would mediate other putative speech-specific phenomenon would require further investigation. The time course for learning auditory categories and specific aspects of the auditory system that makes this possible still need to be explored. This type of research would make it possible to determine what aspects of cue integration are innate versus those that are acquired through general auditory experience and learning. Infant studies are one way to further investigate this question. Early research with infants suggested a maturational component to phonetic cue trading based on findings that infants and children were able to use VOT but not F1 onset frequency information in the discrimination of stop consonants until they reached 5 years of age [66]. Thus it was assumed that phonetic trading relations are dependent on extensive language experience, which would be at odds with the current results. However, later studies showed that infants as young as 2 months are capable of using F1 onset information and do show perceptual equivalence of temporal and spectral information in the discrimination of stop consonants. A study by Miller and Eimas [67] found that when infants are presented with stimuli on a VOT continuum they do show a shift in their category boundaries in response to changes in F1 onset frequency, consistent with the present findings in budgerigars. Researchers have also found that infants show a trading relation between F1 onset frequency and silent gap duration (for a say-stay continuum) similar to earlier findings in adults [68]. These findings in infants combined with the current results in budgerigars point to an innate auditory mechanism behind cue trading that does not rely on speech experience.

In conclusion, the results of this study demonstrate that prior speech experience is not a prerequisite for cue trading in budgerigars. Birds that were never exposed to speech did not differ from birds that were extensively trained to mimic human speech. Therefore, learning to mimic human speech is not required for category formation or trading relations. All bird groups used speech cues in a manner very similar to humans. To the extent that the birds can be used as a model for human speech perception, the current results indicate that speech categories do not depend on articulation, and that trading relations can occur without prior experience with the relevant speech cues.

## Supporting information

S1 File The raw experimental results for individuals across all conditions. (XLSX)

S2 File All stimuli used in these experiments. (ZIP)
