# Correction: Experience with speech sounds is not necessary for cue trading by budgerigars (*Melopsittacus undulatus*)

**DOI:** 10.1371/journal.pone.0189014

**Published:** 2017-11-30

**Authors:** Mary Flaherty, Micheal L. Dent, James R. Sawusch

There is an error in the first sentence in the sixth paragraph of the Discussion section. The correct sentence is: The pattern of responses was similar for budgerigars and humans in that both groups had more /d/ responses in the low F1 onset frequency compared to the high F1 onset frequency and both showed the expected shift in category boundary.
